# Efficacy of multiparametric telemonitoring on respiratory outcomes in elderly people with COPD: a randomized controlled trial

**DOI:** 10.1186/1472-6963-13-82

**Published:** 2013-03-06

**Authors:** Claudio Pedone, Domenica Chiurco, Simone Scarlata, Raffaele Antonelli Incalzi

**Affiliations:** 1 Area di Geriatria. Università Campus Biomedico, Roma, Italy; 2 Fondazione “A. Sordi”, Roma, Italy; 3 Fondazione “S Raffaele - Cittadella della Carità”, Taranto, Italy

**Keywords:** Chronic obstructive pulmonary disease, Aged, Telemonitoring

## Abstract

**Background:**

Chronic obstructive pulmonary disease (COPD) is a highly prevalent condition associated with a high health care resource consumption and health care expenditures, driven mainly by exacerbations-related hospitalizations. Telemedicine has been proposed as a mean for timely detection of exacerbation, but the available evidence is inadequate to provide conclusive information on its efficacy. The aim of this study is to evaluate the efficacy of a telemonitoring system in reducing COPD-related hospitalizations in an elderly population with COPD.

**Methods:**

This is a parallel arms, randomized trial including patients aged 65 or older with COPD in GOLD stages II and III enrolled in a Pulmonary Medicine outpatient facility. Patients were randomly assigned to receive a non-invasive system able to telemonitor vital signs (oxygen saturation, heart rate, near-body temperature, overall physical activity) or standard care, and were followed up for 9 months. The outcome measures were the number of exacerbations and exacerbation-related hospitalization.

**Results:**

Fifty patients were included in the telemonitoring group and 49 in the control group. The incidence rate of respiratory events was 28/100 person/years in the telemonitoring group vs. 42/100 person/years in the control group (incidence rate ratio: 0.67, 95% CI: 0.32 – 1.36). The corresponding figures for hospital admissions where 13/100 person/years and 20/100 person/years, respectively (IRR: 0.66, 95% CI: 0.21 – 1.86).

**Conclusions:**

In our study, COPD patients followed up with the aid of a multiparametric remote monitoring system experienced a lower rate of exacerbations and COPD-related hospitalizations compared to patients followed up using the standard model of care. These results need to be replicated in larger studies before they can be applied to the general COPD population. Trial registration number: NCT01481506 (clinicaltrials.gov). Funding: co-financed by Lazio Region and Intersistemi Inc.

## Background

Chronic obstructive pulmonary disease (COPD) is a highly prevalent condition that is expected to be the third cause of death worldwide by 2020 [1]. It is also associated with important risk of disability with a related very high use of health care resources. It has been reported that hospital care, which is mainly driven by exacerbations, is the main component of health care expenditures, and exacerbations prevention can reduce costs [2]. Comorbidity, mainly cardiovascular, substantially contributes to health care costs [3]. Selected interventions, such as patient’s education and dedicated health programs [4] as well as some pharmacological measures [5], have achieved this objective. Telemedicine has also been tried as a mean for detecting worsening health status and to implement timely interventions [6]. The relevant experience, however, is scarce because the vast majority of the studies are small and lack a control group [7].

Telemonitoring of COPD patients has been so far based upon programmed or on demand vocal interaction or periodical transmission of expiratory peak flow or transcutaneous oxygen saturation (SaO_2_) [8-11]. All these interventions are monodimensional in nature and episodic or periodic in their timing, and have yielded mixed results with respect to cost reduction and quality of life improvement. COPD, mainly in the presence of cardiovascular comorbidity, is multidimensional in its impact on health status [12,13].

Furthermore, older age can modulate the clinical expression of COPD and its exacerbations by increasing the relative weight of non respiratory symptoms such as muscle weakness or dizziness [14]. Thus, a multidimensional approach seems desirable for a comprehensive monitoring of COPD patients, mainly if in the geriatric age, to identify timely changes in health dimensions heralding COPD exacerbations. Such a monitoring system has to be accurate, but also simple, economic and user-friendly in order to be useful in clinical practice.

In the present study we present a randomized, parallel-group trial of a multiparametric remote monitoring system measuring physical activity, cardiac and respiratory rates, near-body temperature and peripheral oxygen saturation.

## Methods

### Study design

This was a single-center, unmasked, randomized trial with 9 months of follow-up of multi-parametric telemonitoring vs. standard care. Patients were recruited among those attending the Pneumology outpatient facilities of the Campus Biomedico University in Rome by a study researcher. Eligibility criteria were age ≥ 65 years and diagnosis of COPD stage II or III according to the GOLD criteria (http://www.goldcopd.org): forced expiratory volume in the first second (FEV1) / forced vital capacity (FVC) <0.7 and 30% <FEV1 percent predicted <80*%*. We excluded patients with a life expectancy <6 months and those with cognitive impairment severe enough to preclude the use of the telemonitoring device. The study protocol was approved by the Ethical Committee at the Campus Biomedico University (#29/2009).

### Intervention

The “SweetAge” monitoring system was developed specifically for this study by a collaborative group of including both research centers and business enterprises (see “Acknowledgements”). It is made up by three main components on the patient’s side: 

1. a wristband that contained the sensors for heart rate, physical activity, near-body temperature, and galvanic skin response. The wristband also contained a bluetooth transmitter;

2. a commercial pulse-oxymeter (Nonin Medical Inc.) connected to a bluetooth transmitter coupled with the wristband;

3. a commercial cellular telephone coupled with the wristband via a bluetooth connection. The telephone was equipped with a software that allowed the reception of the data transmitted by the wristband and acted as a gateway to send the data to the monitoring system.

The system was extensively tested before the beginning of the trial on both healthy controls and COPD patients. The information obtained were compared to those objectively measured, and a satisfactory agreement was obtained. It should be noted that for oxygen saturation the “SweetAge” system used a commercial device, and therefore for this parameter we only tested the dependability of the transmission system.

A dedicated software allowed real-time monitoring of the information gathered by the devices listed above. This monitor system was web-based and accessed through a secure connection using a standard Internet browser. The operation of the components of the patient’s side were mostly automatic: the patients only had to wear the wristband and to turn it on, while keeping the cell phone in the range of the bluetooth transmission. The patient was not aware of wristband operations, except for oxygen saturation: a sound reminded the patient to wear the pulse-oxymeter when the measurement were scheduled.

The system was set up to perform 5 measurement of each parameter every three hours. Oxygen saturation was measured over 1 minute, for the others parameters five measurements of 1 minute each were performed at a sampling rate of 60 Hz. The measurements were averaged by the monitor system and displayed as the mean value to the operator. The individual values were available on demand. The patient could access the data at the moment of measurement, as they were displayed on the telephone’s screen.

The data received were evaluated every day by a physician skilled in the care of respiratory patients. The monitor system displayed an alert when a measurement was outside the predefined range. The limits for these alerts could be customized for the individual patient by the user of the system on the basis of the patient’s specific clinical situation. This system, however, was intended for monitoring only, and the patients were instructed to contact their usual health care provider in case of need.

In case of abnormal readings, the physician contacted the patient to verify whether their symptoms had worsened or new symptoms had arisen. In this event, the patient’s adherence to therapy was checked and, if unsatisfactory, individually tailored interventions promoting adherence were carried up. Otherwise, a diagnosis of exacerbation was made and, on the basis of its severity a office appointment (for mild exacerbations) or a hospital admission was scheduled.

### Outcome

The outcome measures of these study were: number of exacerbations, defined as a sustained worsening of the patient’s condition, from the stable state and beyond normal day-to-day variations, that is acute in onset and necessitates a change in regular medications [15], and number of hospital admissions. All the information were gathered from clinical records, when available, or in person/telephone interview.

### Sample size

We anticipated a mean of 1.5 exacerbation/patient/year [16], corresponding to a risk of 81% of having the event. Allowing for a type I error rate of 5%, we calculated that a sample size of 50 patients per group would provide a 80% power of detecting a 30% reduction of the risk. Participants were allocated to the study groups in a 1:1 ratio using a computer-generated random list of number.

### Analytic approach

The demographic and functional characteristics of the two groups were compared using descriptive statistics and t-test or chi-square test, as appropriate. Physical disability at baseline was evaluated using the basic Activities of Daily Living [17] that investigates 6 domains providing a score ranging from 0 (total disability) to 6 (no disability). Cognitive function was investigated using the Mini-Mental State Examination [18], a scale providing a score ranging from 0 to 30, with lower scores (<24) indicating possible cognitive impairment. The risk for the outcome of interest was evaluated by calculating incidence rates and incidence rate ratios along with 95% confidence intervals. All the analyses were performed using the “intention to treat” approach. The analyses were performed using R Statistical Software version 2.14 for Linux (R Foundation for Statistical Computing, Wien, Austria, 2011).

## Results and discussion

**Figure 1:**
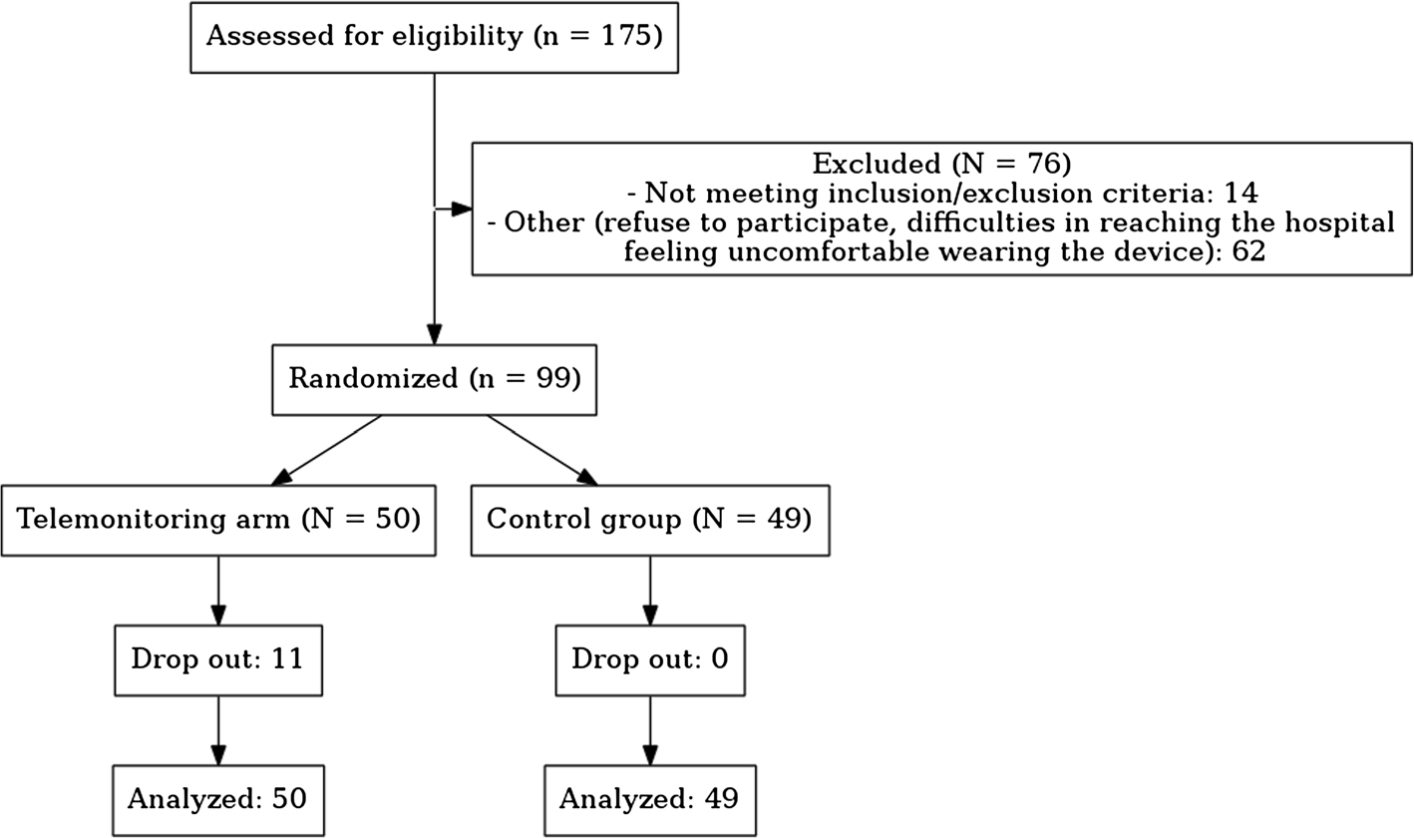
Flow diagram of the study.

The flow diagram of the study is reported in Figure 1. Overall we followed up 99 patients with a mean age of 74 years. Over the follow-up period, 20% of patients dropped out from the study, but were followed for the outcomes of interest. Reasons for drop out were: patient feeling uncomfortable with the wearable device or thinking that they disrupt the daily life rhythm.

About two-thirds (68%) of participants were men. The average FEV1 was 54% of predicted (range 23% – 77%). As shown in Table 1, there were no differences in the main characteristics of participants in the two groups, although controls had a slighly worse performance in instrumental activities of daily living.

**Table 1:** Characteristics of participants

	**SweetAge (N=50)**	**Controls (N=49)**	**P**
Age	74.1 (6.4)	75.4 (6.7)	0.340
Gender (Men)	72%	63%	0.475
FEV1 %	52.5 (14.9)	55.4 (15.8)	0.413
FVC %	78.8 (16.5)	78.5 (16.9)	0.947
GOLD stages			
I	-	-	
II	57.1%	59.5%	0.984
III	42.9%	40.5%	
IV	-	-	
Mean ADL score	5.6 (1.0)	5.1 (1.3)	0.157
MMSE score	28.1 (2.1)	26.7 (4.0)	0.084

On average, the SweetAge equipment sent data for 150 days/patient, with a mean of 4 measurements/day. Considering the days in which the system was actually used, the proportion of days with at least 3 measurements ranged from 0% to 97% (mean: 60%, interquartile range: 50% – 80%). Per protocol, all the events detected by the telemonitoring system triggered at least a telephone call by the study researcher. Only one out of fourteen such calls revealed a self-limiting problem, while in all other cases other actions were undertaken.

**Figure 2:**
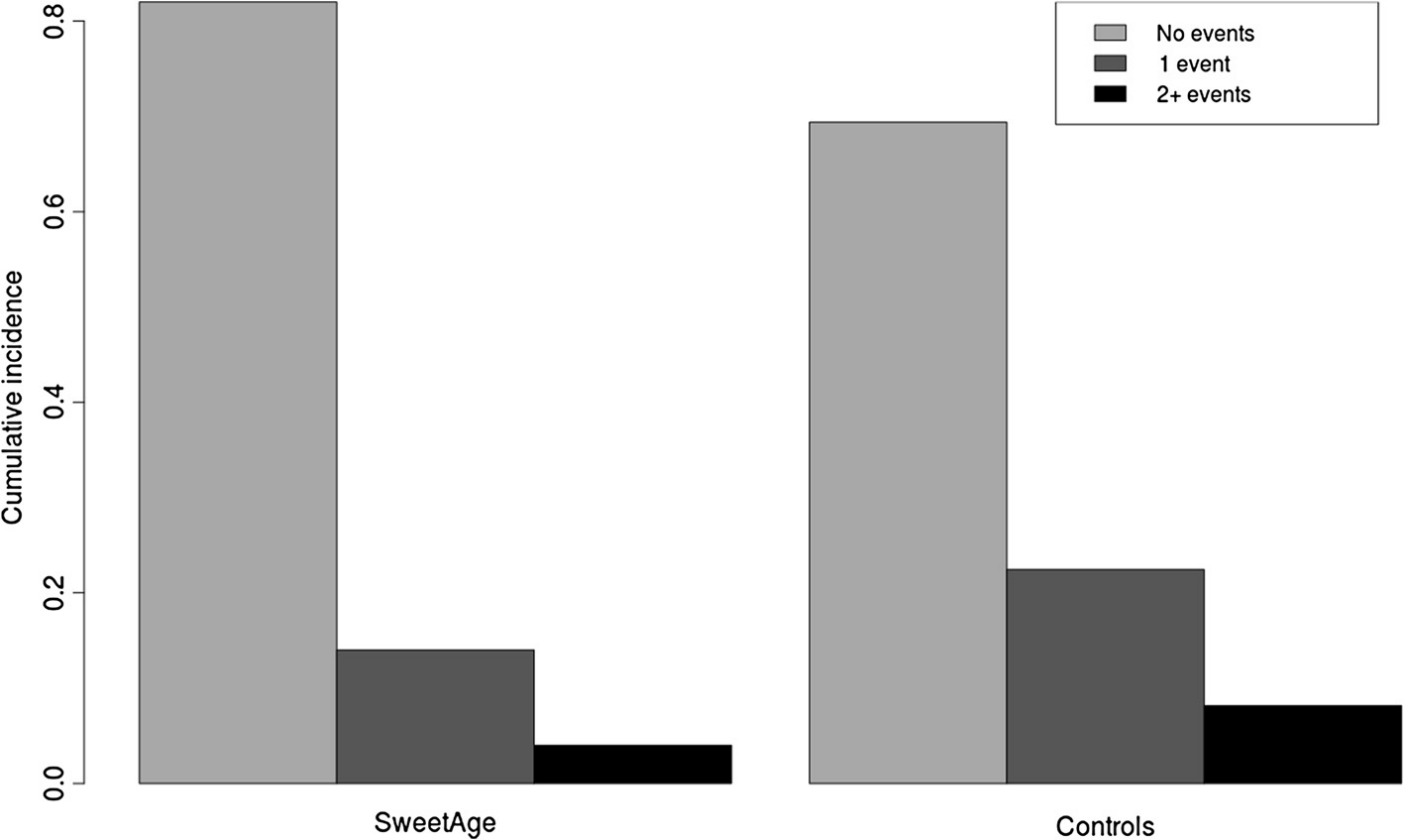
Cumulative incidence of events.

Overall, we observed 19 events in the control group and 13 events in the experimental group. Among controls, 15/49 (31%) participants experienced at least 1 respiratory event, compared to 9/50 (18%) in the SweetAge group. The incidence of multiple events was 8% among controls and 4% in the SweetAge group (Figure 2). In terms of incidence rate, we observed 28 events/100 person year in the SweetAge group and 42 events/100 person year in the control group, with and incidence rate ratio of 0.67 (95% CI: 0.32 - 1.36) (Table 2).

**Table 2:** Respiratory events during follow-up

	**SweetAge (N=50)**	**Controls (N=49)**
Cumulative incidenceof events	18%	31%
Cumulative incidenceof multiple events	4%	8%
Incidence rate	28/100 person-year	42/100 person-year
Incidence rate ratio	0.67 (95% CI: 0.32 – 1.36)	

In both groups, about half of the events have been hospital admissions (46% and 47% in the SweetAge and control group, respectively). The corresponding incidence rate was 13/100 person year in the SweetAge group and 20/100 person year in the control group, with an incidence rate ratio of 0.66 (95% CI: 0.21 - 1.86). The average length of stay was 9.7 days in the SweetAge group and 6.9 days in the control group.

**Figure 3:**
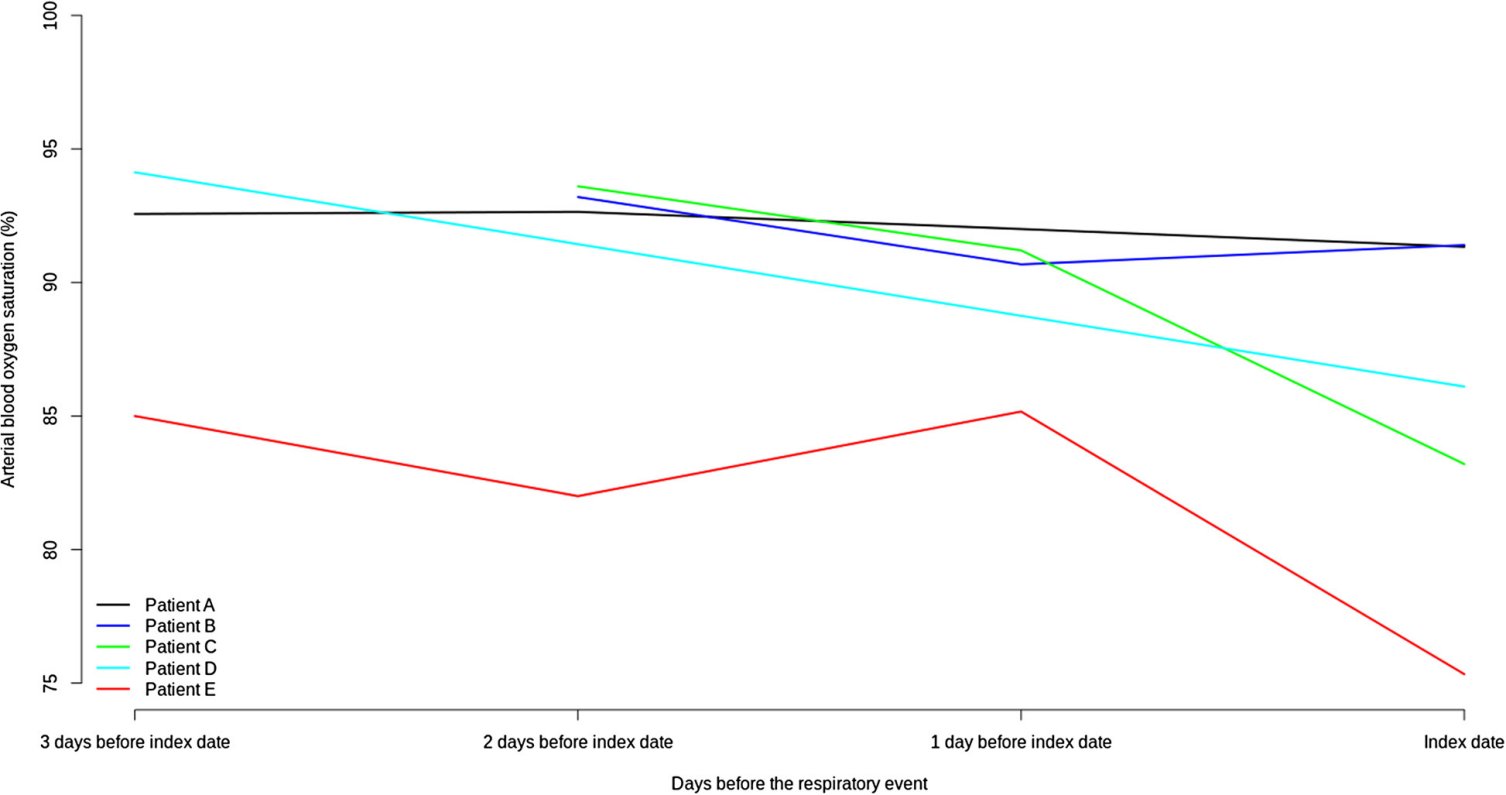
**Arterial oxygen saturation in the three days preceding the respiratory event.** Trend of the arterial oxygen saturation in the three days before a respiratory event. Each line represents data from a single patient.

The SweetAge system revealed a worsening of peripheral oxygen saturation in the days preceding most of the respiratory events (see Figure 3), allowing a timely intervention (e.g. drug regimen modifications) or, in most severe cases, to plan the hospital admission avoiding an emergency room visit.

In our study, COPD patients followed up with the aid of a multiparametric remote monitoring system experienced a lower rate of exacerbations and COPD-related hospitalizations compared to patients followed up using the standard model of care. The presence of a control group and the common operational framework guarantees for the reliability of our data. In addition, we showed that respiratory events can actually be timely detected by a wearable system whose operations are fully automated and require very little skills on the patient’s side.

Previous studies provided controversial results on the possibility of preventing exacerbations through a telehealth approach. A recent review, however, pointed out that only four out of a thousand studies taken into account had a control group [19]. A meta-analysis on this topic [20] concluded that while home telemonitoring and telephone support seem to be effective in reducing the rate of hospitalization and emergency room visits, the evidence on number of hospital days and on mortality are conflicting. In our sample, telemonitoring could cut by 33% the risk for hospitalizations. However, the average length of stay was longer in the study than in the control group. This finding likely is consistent with a lower threshold for hospitalization in the control group, i. e. for a more stringent selection of patients needing hospitalization in the study group. Thus, telemonitoring may be effective promoting home care of less severe COPD exacerbations. This result is in keeping with data showing that a “hospital at home” approach is safe and successful in selected patients [21].

Telephone contacts had a second step role in our telemonitoring design. Indeed, despite some positive evidences [6,22,23], a recent metanalysis of 11 studies shows that telephone based care was associated with greater mortality than usual care [20] in people with COPD. Furthermore, this approach could not benefit patients with heart failure, a condition that, like COPD, is dominated by dyspnea, even in a very large and well conducted trial [24]. It should be noted that dyspnea, the symptom most commonly collected in the telephone interview of cardiac or respiratory patients, is subject to large interindividual variability: non respiratory symptoms such as asthenia, leg discomfort/edema dominate the clinical proportion in a consistent proportion of COPD patients experiencing severe, even life-threatening exacerbations [14]. Furthermore, in patients able to perform physical activity, the ergoreflex, which originates from overworking respiratory muscles, causes peripheral sympathetic overdischarge and, eventually, peripheral vasoconstriction, muscle ischemia and fatigue [25]. As a consequence, patients decrease their already limited activity, which prevents the worsening of dyspnoea and postpones the recognition of the exacerbation. On these bases, we resolved to monitor also non respiratory parameters and to consider the telephonic interview as a second step intervention driven by changes in monitored parameters.

Among the parameters monitored, only oxygen saturation could timely identify COPD exacerbations. This reflects the expected high specificity of O_2_ desaturation: it is very unlikely that a condition other than respiratory exacerbation accounts for this event. However, minor differences in the precritical multiparametric profiles were evident. The relatively short duration of the study and, thus, the limited number of critical events prevented us from verifying whether well defined and repeatable patterns can be identified. A larger study might clarify this issue, which is of practical as well conceptual interest. Indeed, defining different trajectories towards the exacerbation would also shed light on phenotypic variability of COPD.

This study has some limitations. We had a lower incidence of events than expected and consequently the confidence interval around our point estimates were large and we cannot exclude that the better outcomes observed in the telemonitoring group are due to random error. The low rate of events observed may have different causes, including the improved management of COPD in the latest years. Evidence in support of a more aggressive treatment of both COPD and their comorbidities, leading to hospitalization only of more severe exacerbations has been reported by different studies [26,27]. At any rate, despite the reduced statistical power, our results are in line with other showing that remote arterial oxygen saturation monitoring can reduce hospitalizations in people with very severe COPD [28]. Larger studies, however, are needed to confirm the effectiveness of this telemonitoring system. Another potential limitation is that cannot exclude that results were partly due to the education provided to the patients, although cases and controls were given the same basic instructions with regard to perception of changing health status and were offered the same ambulatory support. However, cases more frequently attended the ambulatory to have information on the use of the device, and this might have been a source of difference between groups.

This study also has some methodological and technological strengths. On the methodological side, it was a rigorously designed and conducted randomized trial, which guarantees for reliability of results. On the technological side, at variance form the traditional telemonitoring of COPD patients, monodimensional in nature or, if multidimensional, based on cumbersome technological tools and procedures [20], our telemonitoring system was very easy to use: the patient had to do little more than wearing the device. Moreover, it allowed to monitor the patients at different times of the day and in different conditions (e.g. indoor vs. outdoor, at rest and during exercise, etc.), and was suitable for both chronic monitoring and “on demand” use.

## Conclusions

In conclusion, our data suggest that, compared to standard care, a multiparametric remote monitoring system may reduce COPD exacerbations rates and COPD-related hospitalizations. This favorable experience needs to be replicated in larger populations in order to achieve sufficient statistical power and to derive algorithms able to identify the exacerbation in its “subclinical” stage according to different individual profiles.

Unfortunately, enormous economic efforts are commonly devoted to pharmacologic, but not to technological innovation. This trial adds further evidence to the concept that telemonitoring is worth of implementation and diffusion as a component of the care of COPD.

## Abbreviations

COPD: Chronic Obstructive Pulmonary Disease; GOLD: Global initiative for Obstructive Lung Disease; FEV1: Forced Expiratory Volume in the first second; FVC: Forced Vital Capacity.

## Competing interests

None of the authors has any conflict of interest to disclose in relation to this manuscript. In particular, none of the authors has financial relationship of any kind with Intersistemi SpA or Evolvo srl.

## Authors’ contributions

CP participated in designing the monitoring system and the study. He drafted the manuscript in collaboration with RAI and performed the statiscal analyses. DC participated in recruiting the participants and in the monitoring activity. She revised the manuscript for important intellectual content. SS participated in the follow-up of the control group. He has revised the manuscript for important intellectual content. RAI participated in designing the study. He drafted the manuscript in collaboration with CP and revised it for important intellectual content. All authors read and approved the final manuscript.

## Pre-publication history

The pre-publication history for this paper can be accessed here:

http://www.biomedcentral.com/1472-6963/13/82/prepub
